# MGCPdb, a collective resource for mulberry genome size, chromosome number, and ploidy

**DOI:** 10.48130/forres-0024-0024

**Published:** 2024-08-13

**Authors:** Honghong Wang, Jingchun Liu, Xiaofei Xu, Yan Li, Jianglian Yuan, Yichun Zeng, Yahui Xuan, Sheng Wang, Gang Liu, Ningjia He, Bi Ma

**Affiliations:** 1 State Key Laboratory of Resource Insects, Southwest University, Chongqing 400715, China; 2 School of Computing Technologies, Royal Melbourne Institute of Technology University, Melbourne, Vic 3000, Australia; 3 Sericultural Research Institute, Sichuan Academy of Agricultural Sciences, Nanchong 610066, Sichuan, China

**Keywords:** Flow cytometry, Chromosome count, Mulberry germplasm resources

## Abstract

Mulberry is a critical economic tree with a high diversity of germplasm resources. However, the lack of primary data on genome size, chromosome number, and ploidy for this species limits the exploitation of mulberry resources. In this study, the genome size of 323 mulberry germplasm resources were examined using flow cytometry and chromosome numbers analyzed. The genome sizes ranged from 0.36 to 3.08 Gb, and seven different ploidies of mulberry germplasm resources were identified, with chromosome numbers ranging from 14 to 308. Correlation analysis indicated that genome size (1C) and chromosome number positively correlated. Here, the genome size, chromosome number, and ploidy database MGCPdb (https://mgcpdb.biodb.org) were constructed for mulberry plants, which contains 323 core mulberry germplasm resources and provides raw data of flow cytometry analysis, genome size, and chromosome count. This database is significant and valuable for mulberry genome evolution, polyploidy breeding, and genetic diversity research.

## Introduction

Mulberry (*Morus* spp.) is a perennial deciduous woody plant^[1−3]^, belonging to the order of Rosales, family Moraceae^[4]^ and widely distributed in Asia and Europe, Africa, Oceania, and on the American continent^[5]^. China is rich in mulberry germplasm resources, with thousands of years of mulberry cultivation and breeding history^[6]^. As the birthplace of the global silk industry, it is also an important center for mulberry origin^[7]^. Historically, along with its use as fodder for silkworms, mulberry plays an important role in traditional medicine and nutrition^[8,9]^. Mulberry leaves, branches, roots, and fruits have a variety of medicinal properties. For example, mulberry leaves have a hypoglycemic effect^[10]^, and are frequently used to treat diabetes mellitus^[11,12]^. A previous study proved that mulberry leaves are natural antioxidants^[13]^.

The understanding of the genome size and chromosome ploidy level of a species is a prerequisite for the genomic and biological study of the species and facilitates our comprehension of the evolutionary associations among related species^[14]^. The genome size is a uniquely and vital characteristic of a species and a measure of its complexity. Although numerous mulberry species exist, studies on the mulberry genome size are scarce. Yamanouchi et al. estimated the genome size of diploid mulberries (2n = 2x) ranged from 0.35−0.37 pg, based on a comparison of nuclear DNA content among *Morus*, *Arabidopsis*, and *Oryza*^[15]^. Subsequently, Chang et al. characterized the genome size of 27 mulberry materials of two ploidies levels (2n = 2x and 3x) in Taiwan. The diploid varieties exhibited a genome size range of 0.31 to 0.36 pg, while the triploid variety possessed a genome size of 0.53 pg^[16]^.

The study of the chromosomes of mulberry plants not only provides a fundamental cytological basis for elucidating the classification, origin, evolution, and genetic diversity of mulberry species but is also essential for the mulberry breeding and cultivation of novel varieties^[2]^. Research on the chromosome number of mulberry began with Tahara in 1909^[17]^, and Osawa in 1916^[18]^. In 1964, Wu identified the chromosome number of several diploid mulberries using the compression method^[19]^, which facilitated further studies of mulberry chromosomes in China^[1,3,20−25]^. However, due to the rich mulberry germplasm resources in China and the multiple ploidies of the mulberry chromosomes, only part of the genome and chromosome information could be obtained, impeding the efficient use of mulberry germplasm resources. This makes it necessary to establish a database containing the genome size, chromosome number, and ploidies of mulberry.

In this study, the genome sizes of 323 mulberry germplasm resources were identified, and chromosome numbers were analyzed on 33 representative mulberry resources. Further, the mulberry genome size, chromosome number, and ploidy database MGCPdb were constructed, providing a basis for studies on mulberry genome evolution, breeding, and genetic diversity.

## Materials and methods

### Plant materials

All 323 mulberry germplasm resources were cultivated at the Mulberry Germplasm Nursery in Southwest University, Chongqing, China. From September to October 2022, 323 samples were collected, from each of which three fresh leaves were carefully selected for the preparatory steps leading up to flow cytometry analysis. All information regarding these 323 mulberry resources is presented in Supplemental Table S1. For internal standard references, the young leaves of *Solanum lycopersicum* 'Heinz 1706' and *Zea mays* 'B73' were used, both at one month after sprouting, obtained from the Kunming Institute of Botany, Chinese Academy of Sciences (Kunming, China).

### Genome size determination of mulberry germplasm resources

The configuration of MG^b^ dissociating solution was performed as follows: 45 mmol/L MgCl_2_·6H_2_O, 20 mmol/L MOPS, 30 mmol/L sodium citrate, 1% (W/V) PVP, 0.2% (v/v) Tritonx-100, 10 mmol/L Na_2_EDTA, and 20 μL/mL β-mercaptoethanol were mixed in ultrapure water and the pH adjusted to 7.5 using NaOH.

The samples were placed in 0.8 mL of pre-cooled MG^b^ dissociation solution, and the tissue was rapidly chopped with a sharp blade, transferred to a pre-cooled incubation dish, placed on ice for 10 min, and then filtered through a 40 μm pore size filter to obtain the nucleus suspension. The obtained suspension was stained with a ready-made staining solution (1 mg/mL propidium iodide, PI) mixed with 10 mg/mL RNAase and stored on ice in the dark for 0.5−1 h. The working concentration of the PI staining solution and the RNAase solution was 50 μg/mL^[26−28]^.

The internal standard references were mixed with the samples at relevant proportions. The analysis was carried out using a BD FACScalibur flow cytometer with 488 nm blue light excitation to detect the fluorescence intensity of the radiated light of PI, with 10,000 particles collected in each assay. The coefficient of variation (CV%) was controlled within 5%. Then, the fluorescence intensities of the internal standard references were compared to calculate the DNA content of the samples using the following equation: DNA content of the samples = DNA content of the internal standard references × fluorescence intensity of the samples/fluorescence intensity of the internal standard references. Finally, FlowJo (v10.0) software was employed to process the flow data and generate histograms for further analysis.

### Chromosome count methods for mulberry representative material

Young leaves of the mulberry materials were immersed in 2 mmol/L 8-hydroxyquinoline solution for 4 h at room temperature and protected from light. Then, they were transferred to 1.5-mL Eppendorf tubes, the 8-hydroxyquinoline was rinsed off with Carnall fixative (absolute ethanol and glacial acetic acid were homogeneously mixed in a volume ratio of 3:1)^[29]^, and the leaves were fixed with Carnall fixative until they turned white, followed by storing at 4 °C. The decolorized young leaves were washed three times with bidistilled water for 5 min each, and subsequently, 100 μL 2.5% cellulase and pectinase mixed enzyme solution was added. The mixture was placed in 1.5-mL Eppendorf tubes, followed by enzymatic digestion at 37 °C for 3 h. After enzyme digestion, the adhering enzyme solution was gently washed off with 70% alcohol, using three rinses for 10 min each, and the material was homogenized using a dissecting needle and resuspended with icodextrin. Subsequently, 20 μL of the cell suspension was placed on a slide, dried, and fixed under alcohol. The sample was incubated for 8 h with Giemsa's staining solution, floated with distilled water to remove the floating color of the staining solution, and then inspected under a microscope, selecting clear and dispersed chromosomes. All samples were stored at −20 °C.

### Statistical tests

Linear regression analysis of mulberry genome size and chromosome number were performed using the lm (linear model) function in R (v4.3.2).

### MGCPdb construction

The MGCPdb database was constructed using LAMP (CentOS 7, Apache httpd, MongoDB, and PHP), a comprehensive, efficient, and innovative platform. It consists of open-source software and provides rapid construction. All data and information were stored in MySQL tables, allowing for faster input and retrieval of flow cytometry (FCM) and chromosome number data.

## Results

### Genome size and chromosome numbers of 323 mulberry germplasm resources

**Figure 1:**
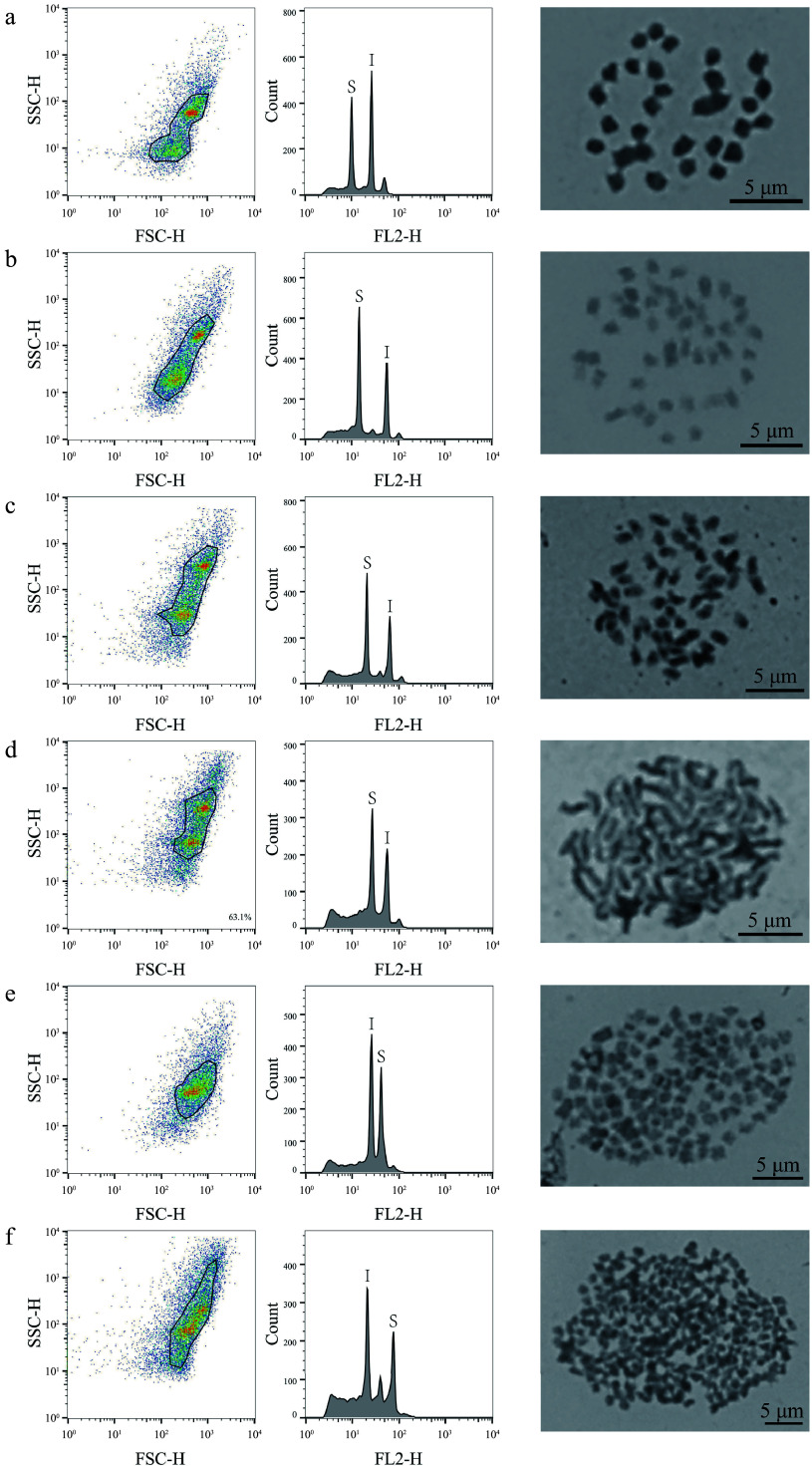
FCM analysis and chromosome count of mulberry germplasm resources. This figure illustrates the results of FCM analysis and chromosome count of representative material of six different mulberry ploidies. The first column shows the FSC-SSC scatter plot of the sample cells, the horizontal axis is the FSC value, which represents the size of the cells, and the vertical axis is the SSC value, which represents the granularity of the cells. The second column of the graph shows the histograms of fluorescence signals FL2-H and cell counts; the horizontal axis represents the DNA fluorescence intensity, the vertical axis represents the number of cells, S denotes the sample material, and I denotes the internal standard reference material. The graphs in the third column are the chromosome count graphs of the corresponding materials, bar = 5 μm; (a) Shanyu1hao, diploid; (b) K34-1, triploid; (c) Ailaoshan5hao, tetraploid; (d) Huai30-2, hexaploid; (e) Pisang2hao, nine-ploidy; and (f) Yaosang, 22-ploidy.

Flow cytometry was used to characterize the genome sizes of 323 mulberry germplasm resources, comparing the results with those obtained for the internal standard reference genomes, which ranged from 0.36 to 3.08 Gb (Supplemental Table S1). In the analysis based on FCM, 320 (99%, 320/323) mulberry materials were identified with only one peak compared to the internal standard references, with genome sizes ranging from 0.36 to 3.08 Gb (Supplemental Table S1; Fig. 1). According to the genome size distribution range of the 320 mulberry germplasm resources, 32 representative mulberry materials were further selected for chromosome count (Supplemental Fig. S1; Fig. 1), which revealed that the 32 representative mulberry materials contained 20 mulberry diploids, five triploids, three tetraploids, two hexaploids, one nine-ploidy, and one 22-ploidy, with corresponding chromosome numbers of 2n = 14, 2n = 28, 2n = 42, 2n = 56, 2n = 84, 2n = 126, and 2n = 308, respectively (Fig. 1, Supplemental Fig. S1; Table 1). In these representative mulberry materials, genome size ranged from 0.36−0.44 Gb for diploid; 0.56−0.62 Gb for triploid; 0.71−0.83 Gb for tetraploid; 0.94−1.10 Gb for hexaploid. The genome size of the nine-ploidy was 1.44 Gb, and that of the 22-ploidy was 3.08 Gb (Table 1).

**Table 1:** FCM analysis and chromosome count of 33 representative mulberry materials.

Material name	Genome size (1C, Gb)	Chromosome number	Ploidy level	Mixed ploids
K27-1	0.38	28	2n = 2x	no
K41-1	0.38	28	2n = 2x	no
K47-1	0.38	28	2n = 2x	no
Aoyu	0.42	28	2n = 2x	no
Baiguo	0.37	28	2n = 2x	no
Chuansang	0.44	14	2n = 2x	no
Guihuami	0.37	28	2n = 2x	no
Huanglusang	0.36	28	2n = 2x	no
Xiang0hao	0.37	28	2n = 2x	no
Yichuanhong	0.37	28	2n = 2x	no
Guoxuan1hao	0.37	28	2n = 2x	no
Hongguo4hao	0.37	28	2n = 2x	no
Luban5hao	0.36	28	2n = 2x	no
Mengjian4hao	0.42	28	2n = 2x	no
Ribentiancheng	0.39	28	2n = 2x	no
Shanyu1hao	0.38	28	2n = 2x	no
Yanjian4hao	0.37	28	2n = 2x	no
Yuansang1hao	0.38	28	2n = 2x	no
Yuansang2hao	0.37	28	2n = 2x	no
Yunsang1hao	0.36	28	2n = 2x	no
K34-1	0.59	42	2n = 3x	no
K36-5	0.62	42	2n = 3x	no
Shidian6hao	0.59	42	2n = 3x	no
Yueshendashi	0.56	42	2n = 3x	no
Taiwanchangguosang	0.58	42	2n = 3x	no
Huasang	0.71	56	2n = 4x	no
Ailaoshan5hao	0.75	56	2n = 4x	no
Ailaoshan9hao	0.83	56	2n = 4x	no
Huai30_2	1.10	84	2n = 6x	no
Baojing7hao	0.94	84	2n = 6x	no
Pisang2hao	1.44	126	2n = 9x	no
Yaosang	3.08	308	2n = 22x	no
Jialing30hao	0.38	28	2n = 2x	yes
Jialing30hao	0.68	56	2n = 4x	yes

**Figure 2:**
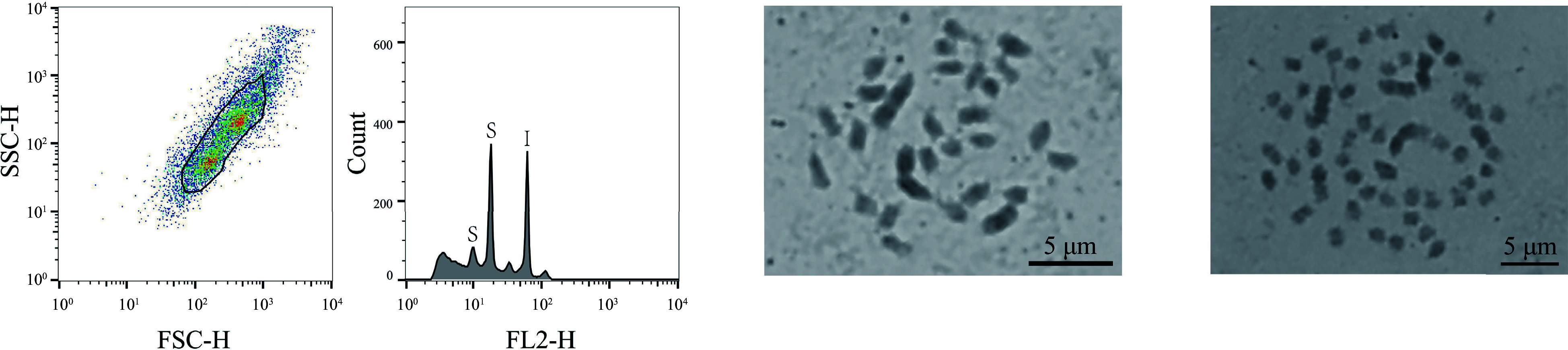
FCM analysis and chromosome count of the haploid mulberry germplasm resource Jialing30hao. The first graph, from left to right, showed the FSC-SSC scatter plot of the sample cells. The horizontal axis is the FSC value, which represents the size of the cells, and the vertical axis is the SSC value, which means the granularity of the cells. The second graph is the histogram of fluorescence signal FL2-H and cell counts. The horizontal axis represents the DNA fluorescence intensity, the vertical axis represents the number of cells, the S of the first peak indicates the sample diploid of Jialing30hao, the second peak of S indicates the tetraploid of sample Jialing30hao, and I indicates the internal standard references *Z. mays*. The third graph shows the results of the chromosome count of the diploid of Jialing30hao; and the fourth graph shows the chromosome count results of the tetraploid Jialing30hao, bar = 5 μm.

Among these 323 mulberry germplasm resources, three were also identified (1%, 3/323) that exhibited mixed-ploidy characteristics (Jialing30hao, Zhe7, Zhe12), with two distinct peaks in comparison with the internal standard references, corresponding to two different ploidy genomes of 0.38 and 0.68 Gb, 0.38 and 0.72 Gb, and 0.39 and 0.74 Gb, respectively, (Supplemental Table S1). Of those, Jialing30hao was selected to conduct a chromosome count. Based on the results, there were two sets of chromosomes in Jialing30hao, with chromosome numbers of 2n = 28 and 2n = 56 (Fig. 2), thus confirming that the material was a mixed ploidy consisting of diploid and tetraploid.

### Positive correlation between genome size and chromosome number in mulberry

**Figure 3:**
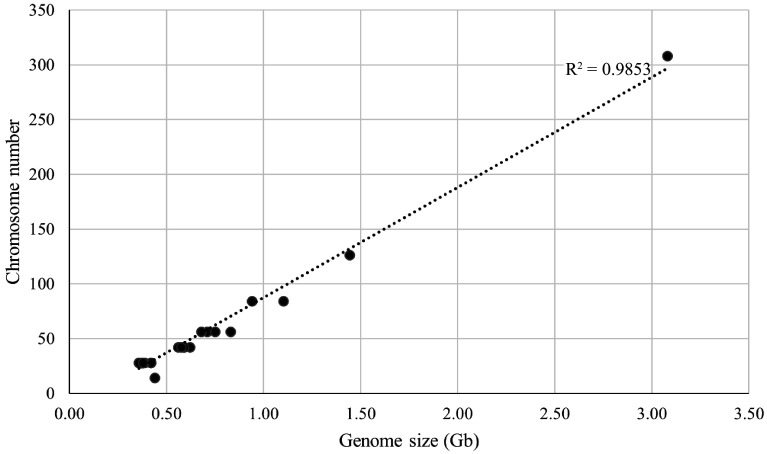
Linear regression analysis between genome size and chromosome number for 33 representative mulberry samples. The horizontal axis is the genome size, and the vertical axis is the chromosome number.

Thirty three representative mulberry materials were selected for chromosome count and the chromosome number and ploidy of these materials were confirmed. These 33 representative mulberry materials contained seven different ploidies (2n = 2x, 3x, 4x, 6x, 9x, 22x, and 2x/4x) with chromosome numbers ranging from 28 to 308 and genome sizes ranging from 0.36 to 3.08 Gb. A linear regression analysis of chromosome number and genome size was further performed (Fig. 3), which revealed a correlation coefficient R^2^ = 0.9853, indicating that these two factors are directly correlated. For example, in Shanyu1hao, the chromosome number was 2n = 28, with a genome size of 0.38 Gb. K34-1, chromosome number was 2n = 42, with a genome size of 0.59 Gb.

### Mulberry genome size, chromosome number, and ploidy database MGCPdb (https://mgcpdb.biodb.org)

**Figure 4:**
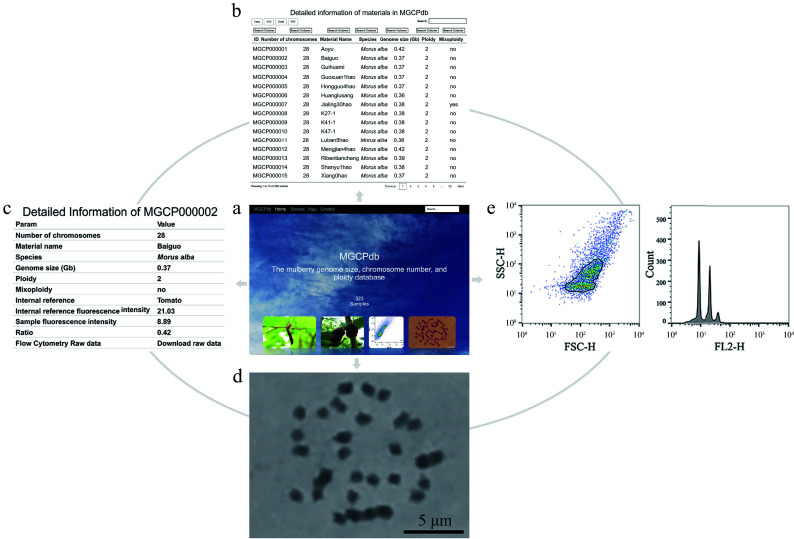
The process of retrieving relevant information in MGCPdb. (a) The menu navigates the MGCPdb database at the top of the page, which contains four main sections: Home, Browse, Help, Contact, and a global search tool in the upper right. (b) The browsing interface for the MGCPdb database, clicking on the four functional areas in the upper left, allows you to directly copy or download the data in CSV, XLSX, and PDF formats, with a personalized search function on top of each column of data. (c) The MGCPdb database provides detailed data tables on genome size, chromosome number, ploidy, and other related information of every sample. (d) and (e) correspond to the sample analysis graph's chromosome count and FCM results.

For more convenience, the genome size, chromosome number, and ploidy database MGCPdb were constructed for mulberry by summarizing the information of all materials investigated in this study (Fig. 4a). This database offers both global and targeted search options (Supplemental Fig. S2), allowing researchers to quickly search (Fig. 4b) and browse (Fig. 4c) detailed information on the material of interest, including information on genome size, chromosome number, ploidy, chromosome diagram (Fig. 4d), and flow cytometry analysis results (Fig. 4e). In addition, there is the option to directly copy or download data in CSV, XLSX, and PDF formats (Supplemental Fig. S2). Raw flow cytometry results for all materials are available for download in MGCPdb, making it easy for users to analyze the data locally (Fig. 4c).

## Discussion

### Mulberry has abundant polyploid resources

Using FCM, the plant genome size can be identified accurately, efficiently, rapidly, and in high-throughput screening. As early as 1998, Doležel et al. determined the genome size ranges of nine plant species using flow cytometry^[30]^. Later, Yamanouchi et al. estimated the genome size ranges of mulberry diploids (2n = 2x) by comparing the nuclear DNA content of *Morus*, *Arabidopsis*, and *Oryza*^[15]^. Chang et al. characterized the genome size ranges of 27 mulberry materials of two ploidy (2n = 2x and 3x)^[16]^. Recently, Gnanesh et al. examined the genome size of four different ploidies (2n = 2x, 3x, 4x, and 6x), totaling 157 mulberry germplasm resources, and carried out chromosome count of 12 mulberry materials^[31]^. Kruthika et al. conducted genome size assays of six different ploidies (2n = 2x, 2x + 2, 3x, 4x, 6x, and 22x), totaling 162 mulberry germplasm resources^[32]^.

The present study included seven mulberry types with different ploidies (2n = 2x, 3x, 4x, 6x, 9x, 22x, and 2x/4x) among the 323 mulberry germplasm resources determined. So far, this is the most quantitative and ploidy-comprehensive study covering the most significant number of mulberry germplasm resources^[31−33]^. In plants, polyploidization is an important driving force for the rapid evolution of plant genomes and the formation of species diversity, and polyploid plants are widely distributed^[34]^. The present results further confirm that the species diversity of mulberry is based on a rich diversity of germplasm resources, and this information is of great significance to the breeding and genetic diversity studies of mulberry germplasm resources.

### Mulberry genome size and chromosome number are positively correlated

The results of this study, combined with flow cytometry and chromosome numbers indicate a positive correlation between genome size and chromosome number in mulberry. This is in agreement with previous findings on *Vaccinium* (section *Cyanococcus*)^[35]^, *Ipomoea*^[36]^, and *Rubus*^[37]^, in which the DNA content increases with chromosome numbers. However, it should be emphasized that despite the positive correlation between genome size and chromosome number in mulberry plants, there is no whole-ploidy variation. For example, chromosome number ranges from 28 to 56, and the genome size rises roughly by 1.5. This may be due to the presence of multiple mitotic grains in the mulberry genome^[25]^, the complexity of mulberry genome variation, and the phenomena of chromosome rearrangements and chromosome fusion breaks, which make the mechanism of variation extremely complex^[25,38,39]^. Further, the polyploid *Brassica napus* is undergoing diploidization^[40]^, which may also occur in the high-ploidy resources of mulberry.

### Mulberry genome size, chromosome number, and ploidy database MGCPdb

Genome size is a crucial biodiversity trait of biological and evolutionary significance^[41,42]^, and understanding information such as genome size and chromosome ploidy of a species is a prerequisite for conducting plant genomics research. In 2002, Leitch & Bennett released the Plant DNA C-values database (release 1)^[43]^, which was updated six times until 2019, when Pellicer & Leitch released the newest Plant DNA C-values database (release 7.1)^[44]^, which contains genome size data for 12,273 species. In 2011, Garnatje et al. developed a genome size in the Asteraceae database (GSAD), and in 2018^[45]^, Virales et al. released the latest GSAD (release 3.0), which contains the genome size of 2018,40 species^[46]^. However, only the Plant DNA C-values database (release 7.1) includes data for *Morus alba*.

For convenience and to enhance data use efficiency, the genome size, chromosome number, and ploidy database MGCPdb was constructed for mulberry. The database contains 323 core mulberry germplasm resources and provides raw data for simultaneous FCM analysis, genome size, and chromosome count. The constructed database is open to any user and permanently accessible, user-friendly, and a dynamically updated database. The research will be continued accordingly and more information added about mulberry germplasm resources. In this sense, the MGCPdb is a professional, integrative, and comprehensive data innovation platform that provides valuable data resources for plant genome evolution, mulberry polyploid breeding, and mulberry species genetic diversity research.

## Conclusions

In this study, the genome sizes of 323 diverse mulberry germplasm resources were evaluated using flow cytometry, and chromosome count was carried out on 33 mulberry resources to determine their chromosome number and ploidy. Finally, all data were meticulously integrated and used to construct the mulberry genome size, chromosome number, and ploidy database (MGCPdb). The database provides an essential reference for studying mulberry genome evolution, polyploid breeding, and genetic diversity.

## SUPPLEMENTARY DATA

Supplementary data to this article can be found online.

## Data Availability

All data generated or analyzed during this study are included in this published article and its supplementary information files.
