# Supplementary material for: MGCPdb, a collective resource for mulberry genome size, chromosome number, and ploidy
Source: For Res (Fayettev). 2024 Aug 13;4:e027. doi: 10.48130/forres-0024-0024 (PMC11524241; doi:10.48130/forres-0024-0024)
Supplement: Supplementary file 1 — Supplementary data to this article can be found online. [file forres-0024-0024-S1.zip › 10.48130_forres-0024-0024-Suppl-FigureS2.pdf]

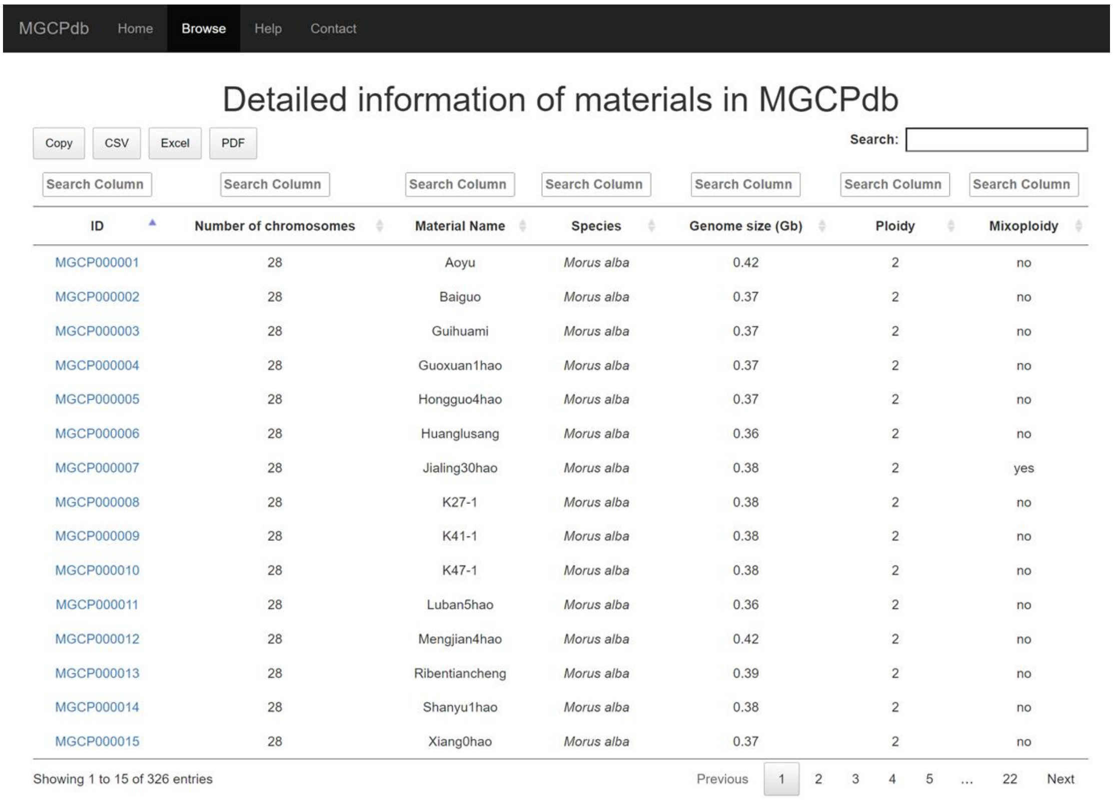

MGCPdb Home Browse Help Contact

Detailed information of materials in MGCPdb

Copy CSV Excel PDF Search:

ID	Number of chromosomes	Material Name	Species	Genome size (Gb)	Ploidy	Mixoploidy
MGCP000001	28	Aoyu	*Morus alba*	0.42	2	no
MGCP000002	28	Baiguo	*Morus alba*	0.37	2	no
MGCP000003	28	Guihuami	*Morus alba*	0.37	2	no
MGCP000004	28	Guoxuan1hao	*Morus alba*	0.37	2	no
MGCP000005	28	Hongguo4hao	*Morus alba*	0.37	2	no
MGCP000006	28	Huanglusang	*Morus alba*	0.36	2	no
MGCP000007	28	Jialing30hao	*Morus alba*	0.38	2	yes
MGCP000008	28	K27-1	*Morus alba*	0.38	2	no
MGCP000009	28	K41-1	*Morus alba*	0.38	2	no
MGCP000010	28	K47-1	*Morus alba*	0.38	2	no
MGCP000011	28	Luban5hao	*Morus alba*	0.36	2	no
MGCP000012	28	Mengjian4hao	*Morus alba*	0.42	2	no
MGCP000013	28	Ribentiancheng	*Morus alba*	0.39	2	no
MGCP000014	28	Shanyu1hao	*Morus alba*	0.38	2	no
MGCP000015	28	Xiang0hao	*Morus alba*	0.37	2	no

Showing 1 to 15 of 326 entries Previous 1 2 3 4 5 … 22 Next

Figure S2. The browsing interface for the MGCPdb database.

The browsing interface has a global search tool in the upper right. Clicking on the four functional areas in the upper left allows you to directly copy or download the data in CSV, XLSX, and PDF formats, with a personalized search function on top of each column of data.
