# Supplementary figures and images for: MGCPdb, a collective resource for mulberry genome size, chromosome number, and ploidy
Source: For Res (Fayettev). 2024 Aug 13;4:e027. doi: 10.48130/forres-0024-0024 (PMC11524241; doi:10.48130/forres-0024-0024)

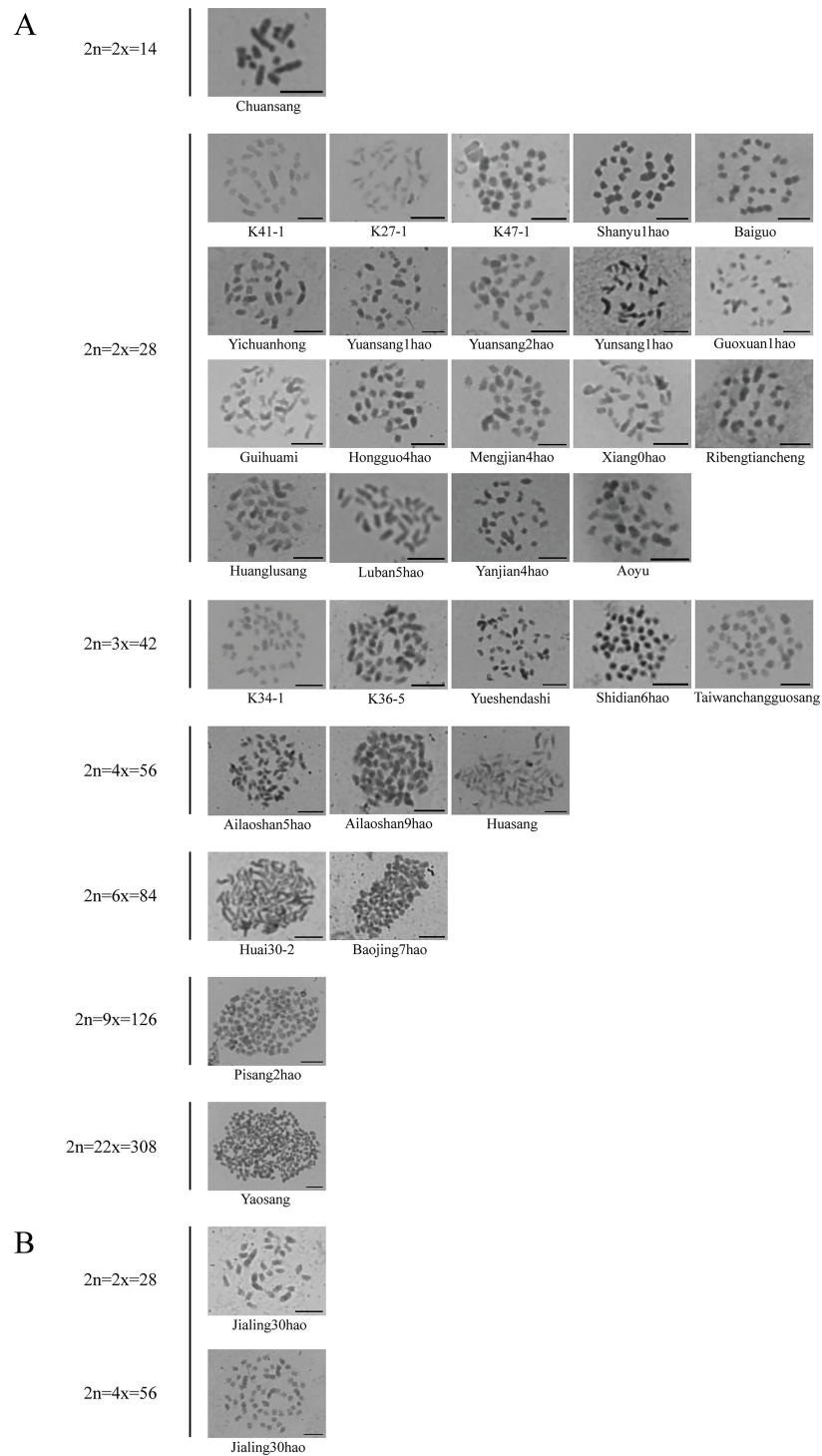

**Figure S1. Chromosome count of mulberry germplasm resources.**  
333 (bar=5  $\mu$ m)

Supplement: Supplementary file 1 — Supplementary data to this article can be found online. [file forres-0024-0024-S1.zip › 10.48130_forres-0024-0024-Suppl-FigureS1.pdf]
